# Siblings in the ICU: Keeping Endemic Mycoses on the Differential

**DOI:** 10.1155/crpe/8693571

**Published:** 2025-08-29

**Authors:** Meaghan Reaney, Brittany Player, Ramya Billa, Claudia P. Vicetti Miguel, Daiva Parakininkas, Charles B. Rothschild

**Affiliations:** ^1^Department of Pediatrics, Section of Critical Care, Medical College of Wisconsin, Milwaukee, Wisconsin, USA; ^2^Department of Pediatrics, Section of Infectious Disease, Medical College of Wisconsin, Milwaukee, Wisconsin, USA; ^3^Department of Pediatrics, The University of Texas Southwestern, Dallas, Texas, USA

## Abstract

Blastomycosis is a rare fungal infection caused by the inhalation of *Blastomyces dermatitidis* spores. Infection with this fungus can impact nearly every organ system, though pulmonary disease is the most common. Presentations of pulmonary blastomycosis are highly variable, ranging from clinically asymptomatic to severe respiratory failure requiring intensive care. This case series describes the clinical presentation, diagnostic challenges, management, and outcomes of two siblings with severe pulmonary blastomycosis that progressed to pediatric acute respiratory distress syndrome requiring mechanical ventilation and venovenous extracorporeal membrane oxygenation (VV-ECMO). Despite being relatively uncommon, blastomycosis should be considered in patients with respiratory symptoms not responding to empiric antibacterial therapy, particularly in endemic regions. Early diagnosis and prompt initiation of appropriate antifungal therapy are crucial for favorable outcomes. Additionally, early initiation of ECMO for severe pulmonary blastomycosis may be beneficial in temporizing to allow time for sufficient response to antifungal therapy.

## 1. Introduction

Blastomycosis is a fungal infection caused by *Blastomyces dermatitidis*, which primarily affects the respiratory system after inhalation of fungal spores from the environment. *Blastomyces dermatitidis* is endemic to the Ohio and Mississippi River Valley, Great Lakes, and southern parts of the United States. It can involve nearly every organ system, commonly presenting with cutaneous, pulmonary, genitourinary, neurologic, and musculoskeletal symptoms. Pulmonary blastomycosis is the most common manifestation, ranging from asymptomatic pulmonary infiltrates in up to 50% of cases to, rarely, complete respiratory or circulatory collapse resulting in mechanical ventilation, extracorporeal membrane oxygenation (ECMO), or death [1, 2]. Devastating pulmonary or disseminated disease can occur in both immunocompetent and immunocompromised patients [3]. While ECMO has been noted in adult literature as a rescue therapy for acute respiratory failure secondary to blastomycosis, literature for the use of ECMO for this diagnosis in pediatric patients is limited [4–8]. This case report details the clinical course, diagnostic challenges, and management of two cases of severe pulmonary blastomycosis in a set of siblings, each requiring critical care interventions.

## 2. Case Descriptions

### 2.1. Case 1

A 9-year-old female with Prader–Willi syndrome, hypothyroidism, and no pre-existing pulmonary disease presented to an emergency department (ED) with a 6-day history of fatigue and worsening right flank pain. At the time of presentation, she was found to have a temperature of 98.3°F, heart rate (HR) of 133, respiratory rate (RR) of 45, oxygen saturation (SpO_2_) of 96%, and blood pressure (BP) of 141/55.

**Figure 1:**
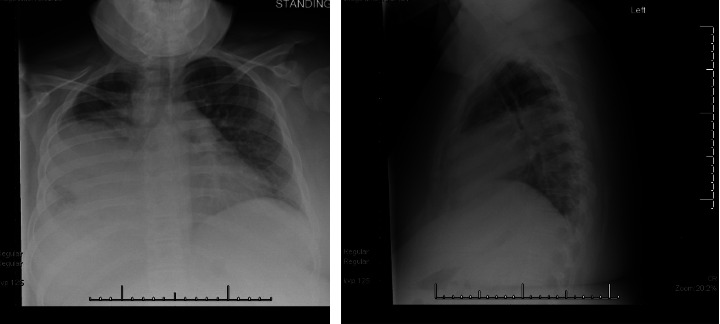
Anterior-posterior and lateral chest X-ray obtained at time of transfer from OSH for Case 1.

Her initial evaluation included serum glucose of 203, hemoglobin A1c (HbA1c) of 8.1%, venous blood gas with pH of 7.37, pCO_2_ of 49, pO_2_ of 38, a complete blood count that demonstrated a white blood cell count of 13.9 k/μL, lactate of 2.6 mmol/L, procalcitonin within normal limits, a negative SARS-CoV-2 nucleic acid amplification test (NAAT) from a nasopharyngeal (NP) swab, and a chest X-ray with a focal consolidation in the right lower lobe. While in the ED, she was started on oxygen via nasal cannula, though it is unclear from records if this was due to hypoxia or increased work of breathing. She was given ceftriaxone and azithromycin after a blood culture was obtained and was subsequently admitted to the community hospital with presumed bacterial pneumonia. On hospital day five, she developed a worsening cough, respiratory distress, and fever with a T max of 102°F. A C-reactive protein (CRP) at that time was 38.5 mg/dL (reference range 0–1.0 mg/dL), and a repeat chest X-ray demonstrated a new right pleural effusion. Her antimicrobials were transitioned to vancomycin monotherapy, and she was transferred to the acute care floor of a free-standing children's hospital. Upon transfer, vital signs were HR of 145, RR of 38, temperature of 37.4°F, BP of 120/86, and SpO_2_ was 91% on 4L via OxyMask with a BMI of 41 kg/m^2^. She was noted to have diminished breath sounds in the middle and lower lung fields, and a chest X-ray was repeated (Figure 1). Antibiotics were broadened to ceftriaxone and vancomycin for empiric treatment of complicated pneumonia. After 24 h without clinical improvement, azithromycin was added. Twelve hours later she required escalated respiratory support and was transferred to the pediatric intensive care unit (PICU) for noninvasive positive pressure ventilation with continuous positive airway pressure (CPAP). Less than 12 h after PICU admission, she required intubation and initiation of vasoactive support. Additional work-up at the time of her clinical decompensation included *Blastomyces* and *Histoplasma* urine antigen, repeat blood culture, urine culture, and culture of endotracheal aspirate. Her empiric antimicrobials were broadened to include enteral fluconazole. However, the patient remained persistently febrile at 105°F. A detailed exposure history was obtained, revealing that the family owned and occasionally traveled to their cannabis farm. The child had direct exposure to a pet rat, wild rabbits, and a reportedly ill cat with significant skin lesions. The family also reported that the child participated in a recent camping trip, swam in freshwater lakes, and had exposure to ticks. At that time, the diagnostic work-up was broadened to include a respiratory pathogen panel by polymerase chain reaction (PCR), fungal and mycobacterial cultures of endotracheal aspirate, Cytomegalovirus (CMV) and Epstein–Barr virus (EBV) panels, as well as serologies for *Coxiella*, *Blastomyces*, *Histoplasma*, *Toxoplasma*, *Bartonella*, and *Chlamydophila psittaci*. The child's antimicrobials were empirically broadened to vancomycin, cefepime, levofloxacin, and liposomal amphotericin B.

**Figure 2:**
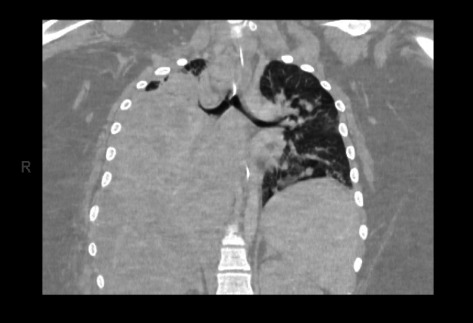
Contrast-enhanced tomography of the chest demonstrating right lung consolidation in Case 1.

Given the progressive nature and severity of her pneumonia, a chest CT with contrast was performed (Figure 2) which showed consolidation of the right lung with a pleural effusion and patchy nodular opacities of the left upper and lower lung in addition to reactive mediastinal and supraclavicular adenopathy. A developing right lung empyema was also identified, and a right-sided chest tube was placed. Bacterial, fungal, and mycobacterial cultures of the pleural fluid were obtained. Bronchoscopy was performed and revealed significant edema throughout with near occlusion of the right distal bronchi and significant compression of the bronchi throughout. A bronchoalveolar lavage (BAL) was performed and sent for testing.

**Figure 3:**
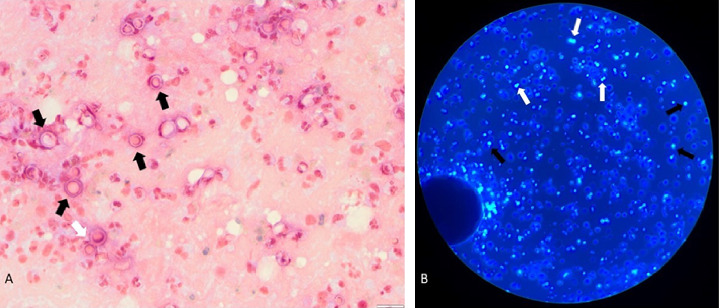
Large yeast (black arrows), some with broad-based budding (white arrows) are demonstrated by (A) gram stain and (B) Calcofluor-white stain of endotracheal aspirate from Case 1.

A Calcofluor stain of the BAL and endotracheal aspirate (Figure 3) revealed broad based budding yeast, most consistent with *Blastomyces* species which was confirmed with isolation of *Blastomyces dermatitidis* by culture 16 days after its initial collection. Three days after initial collection, *Blastomyces* urine antigen also returned positive. Direct florescent antibody (DFA) testing of BAL sample was also *Legionella pneumophila* positive. NAAT for *Legionella pneumophila* from the BAL sample was negative as was *Legionella* antigen in urine testing, suggesting a false positive DFA for Legionella. However, she ultimately completed a full course of levofloxacin while the work-up continued. Full body and brain MRI ruled out disseminated blastomycosis. She was weaned off vasoactive support over 6 days and extubated to BiPAP after 19 days of mechanical ventilation. During the first few days of admission, her glucose levels were > 300 mg/dL and HbA1c was 8.1%, consistent with new onset insulin-dependent type 2 diabetes. She was started on nightly insulin glargine, insulin lispro for mealtime correction and daily metformin. She was ultimately discharged home on 2L nasal cannula during sleep with a plan for outpatient sleep study and itraconazole for a planned 12-month of antifungal therapy.

### 2.2. Case 2

**Figure 4:**
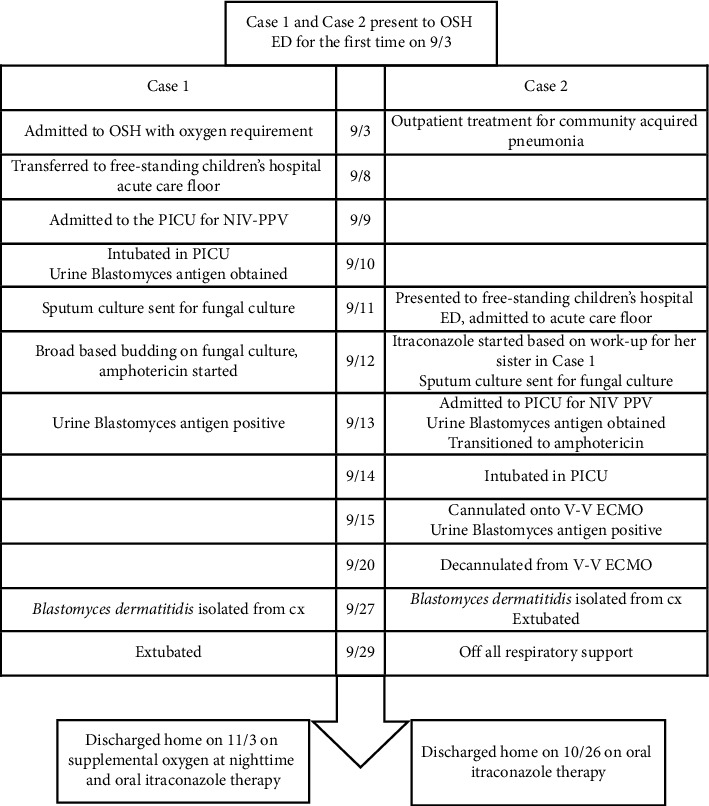
Timeline of events in the courses of Case 1 and Case 2. OSH  = outside hospital; ED = emergency department; PICU = pediatric intensive care unit; NIV-PPV = noninvasive positive pressure ventilation; VV-ECMO = venovenous extracorporeal membrane oxygenation; cx = culture.

A 11-year-old female sister of index Case 1 with no significant past medical history who initially presented to their local ED on the same day as her sister with symptoms of left flank pain, cough, and congestion (Figure 4). Her initial vital signs included BP 128/84, HR 112, RR 20, SpO_2_ 99%, and temperature 98.7°F. On exam, she had a normal respiratory effort with rales in her left lower lung field. Initial evaluation included SARS-CoV-2 NAAT from a NP swab which was negative and a chest X-ray showing a retrocardiac opacity. She was diagnosed with bacterial pneumonia, and due to a reported history of amoxicillin allergy, she was prescribed cefuroxime and discharged home. She returned to the ED 2 days later with a worsening cough and left-sided chest pain. COVID-19, influenza, and respiratory syncytial virus testing from a nasopharyngeal swab was negative at that time, and a repeat chest X-ray demonstrated right upper and left upper and lower lobe consolidations with probable left effusion. Antibiotic coverage was changed to cefdinir and azithromycin. Three days later, at the primary care physician follow-up, cefdinir and azithromycin were discontinued, and she was started on levofloxacin monotherapy. For the next two days, symptoms continued to worsen with a new onset of fever (*T*_max_ 101°F), fatigue, and new conversational and exertional dyspnea as well as orthopnea requiring her to sleep upright. At that point, 9 days after the initial symptom onset and the day after her sister was intubated in the pediatric intensive care unit, her family brought her to a free-standing children's hospital ED for further evaluation.

**Figure 5:**
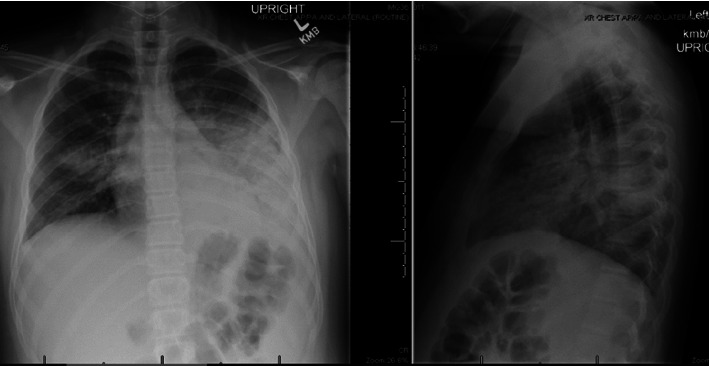
Anterior-posterior and lateral chest X-ray obtained at time of transfer from OSH for Case 2.

At the time of presentation to the tertiary hospital ED, she had an HR of 126, BP of 123/62, RR of 38, temperature of 99.5°F with SpO_2_ of 95% on room air, and a BMI of 25 kg/m^2^; a repeat chest X-ray was obtained (Figure 5). Initially, she was admitted to the pediatric inpatient floor and was placed on 2 L/min OxyMask for oxygen saturations in the upper 80s. She was started on an empiric antibiotic regimen of ceftriaxone and clindamycin. Initial lab evaluation included a complete blood count with a white blood cell count of 14.9 k/μL, procalcitonin of 0.28 ng/mL, CRP of 33.1 mg/dL, glucose of 204 mg/dL, and HbA1c of 8.2%. A respiratory viral PCR panel and blood culture were sent. An exposure history was obtained which revealed direct contact with a pet rat, wild rabbits, an ill cat, and a history of camping with ticks and freshwater exposures, like her sibling (Case 1). Further work-up was then obtained including EBV and CMV panels, Quantiferon gold, and serologies for *Histoplasma*, *Blastomyces*, *Bartonella*, *Chlamydophila psittaci,* and *Francisella tularensis.* Sputum bacterial and fungal cultures were sent. Urine culture and antigens for *Legionella*, *Histoplasma*, and *Blastomyces* were also obtained. Her antimicrobial regimen was broadened to include gentamicin, doxycycline, and itraconazole, given her sister's culture data suggesting endemic mycoses. Two days after admission, her sputum stain was positive for broad-based budding yeast, and her respiratory status worsened with persistent hypoxia in the mid-80s. Antifungal therapy was changed to liposomal amphotericin B. She was transferred to the PICU, where she rapidly progressed to respiratory failure requiring intubation and developed catecholamine-resistant septic shock requiring vasopressor support. She had poor oxygenation requiring, very high ventilator settings. Despite the transition to high-frequency oscillatory ventilation (HFOV), she continued to oxygenate poorly and was semielectively cannulated onto venovenous ECMO (VV-ECMO) on PICU Day 2. During this time, *Blastomyces* urine antigen resulted as positive 2 days after initial collection. She remained on VV-ECMO until decannulation on PICU Day 6, was eventually extubated to nasal cannula on PICU Day 13, and quickly weaned off all respiratory support. Sputum culture resulted in *Blastomyces dermatitidis* 15 days after initial collection. Her course was complicated by a central line-provoked right iliac to popliteal vein thrombus for which she received 8 weeks of anticoagulation. She also developed melena during her hospital course, leading to an EGD that revealed duodenal bulb ulcerations treated with 3 months of a proton-pump inhibitor. HbA1c was noted to be 8.2%, and she was diagnosed with a new onset insulin-dependent type 2 diabetes, for which she was started on nightly insulin glargine and daily metformin. Given the new onset of diabetes in these sisters, monogenic diabetes was considered. The family was approached with this suspicion but declined further testing. She was discharged home on itraconazole monotherapy for a planned 12 months of therapy.

## 3. Discussion

These two cases of severe pneumonia were refractory to empiric antibacterial therapy and resulted in rapid, progressive respiratory decline. Blastomycosis-related acute respiratory distress syndrome (ARDS) is often not considered until late in the disease course, and a delay in diagnosis can lead to poor outcomes [9]. The cases described here are unique, with Case 2 representing the youngest patients with severe pulmonary blastomycosis to survive ECMO. Previous cases involving pediatric patients treated with ECMO for blastomycosis-related pARDS resulted in death [7, 8, 10]. However, in the current report, both patients with blastomycosis-related pARDS survived to hospital discharge, likely due to early initiation of empiric antifungal therapy and prompt respiratory supportive care, including the early initiation of ECMO in Case 2. This highlights the importance of a high index of suspicion for fungal etiologies, particularly in endemic areas [3, 9]. While blastomycosis manifests as a pulmonary infection in 90% of cases, its clinical presentation can mimic other respiratory infections, ranging from acute self-limited pneumonia to chronic pneumonia or rapidly progressing respiratory failure, often making the diagnosis challenging [2, 11]. Radiographic findings, such as infiltrates, nodules, and cavitary lesions, are common and nonspecific, whereas microscopic examination of respiratory specimens, serology, antigen testing, and fungal cultures aid in establishing the diagnosis. In general, urine antigen testing is the most sensitive diagnostic assay (80% sensitivity for all cases and up to 88% for pulmonary cases), followed by KOH/Calcofluor smear of respiratory specimens which has about 47% sensitivity [12]. In the cases presented here, urine antigen as well as smear and culture of respiratory specimens (tracheal aspirate and BAL for Case 1 and sputum for Case 2) were performed; each ultimately confirmed the diagnosis of blastomycosis. The advantage of obtaining respiratory specimens for KOH/Calcofluor smear is the likely faster turnaround time when compared to antigen testing and the possibility of initiating treatment earlier. In a study published in 2021, no difference was found in the sensitivity of fungal smears between sputum and lower respiratory tract specimens, suggesting the utility of sputum and tracheal aspirates to aid in faster diagnosis if BAL cannot be performed in a timely manner or is not otherwise indicated [13].

Various risk factors contribute to the development of blastomycosis, including living in endemic areas or having typical environmental exposures. However, cases have been reported in individuals without specific known exposures, and a wide range of disease severity exists [1, 2, 9]. Risk factors for severe disease include immunosuppression, obesity, and exposure to high inoculum of *Blastomyces dermatitidis* conidia [14]. In the reported cases, their new diagnosis of diabetes likely contributed to the severity of their disease. It has been previously reported that diabetes is associated with a higher risk of progression to respiratory failure and ARDS in pulmonary blastomycosis. A case series of adults in Indiana reported a strong association between having diabetes and admission to ICU (OR 2.9; 95% CI 1.04–8.5) [15]. Two additional studies from the same group in Canada demonstrated that diabetes was common (44%) in adult patients that progressed to ARDS, with a greater association of diabetes in patients admitted to the ICU (OR 3.1; 95% CI 1.3–7.4) [4, 16]. The concomitant diagnosis of diabetes and severe blastomycosis in both sisters is intriguing, and the nature of the association is incompletely understood. It seems most likely that both patients had undiagnosed diabetes which left them susceptible to more severe disease. We suspect a genetic predisposition, such as monogenic diabetes, but testing was declined. In general, in cases of severe blastomycosis infection of unclear etiology, care providers should also maintain an index of suspicion for previously undiagnosed risk factors for severe disease such as diabetes or primary immunodeficiency syndromes [12].

Mortality rates for blastomycosis range from 6% to 9% in adults and 3% to 4% in pediatric patients and are associated with delay to diagnosis [10]. Non-Caucasian racial and ethnic groups, especially African American children, have an increased incidence of moderate or severe disease and higher mortality rates [9, 17]. In one study, Hispanic, Asian, and American Indian and Alaska Native white patients had a 2-3 times higher risk of hospitalization than non-Hispanic white patients. This same study demonstrated a higher likelihood of dying in patients hospitalized due to blastomycosis (10%) as compared to nonhospitalized patients (1%) [17]. Mortality is further substantially increased in patients who require ICU admission (35%) and in those requiring mechanical ventilation (40%) [2, 15, 18]. ARDS secondary to blastomycosis carries a mortality rate of 40%–99% [5, 16, 19, 20]. Many studies have reported an association between elevated BMI and disease severity or outcomes [21–24]. Both patients in this case report demonstrated an elevated BMI for age, specifically > 99^th^ percentile for age in Case 1 at 41 kg/m^2^ and > 95^th^ percentile for age in Case 2 at 25 kg/m^2^. It is conceivable that this patient characteristic could have predisposed them to a more severe disease course.

The initiation of empiric antifungal therapy in cases of suspected blastomycosis while awaiting confirmatory diagnosis is important to reduce the mortality risk. Liposomal amphotericin B is indicated for empiric treatment of severe pulmonary and disseminated disease, and this was initiated for both patients in this report when blastomycosis was suspected. Itraconazole is used as stepdown treatment and continued for a minimum of 12 months. In our report, both patients were successfully transitioned to oral itraconazole after clinical improvement and were discharged home on this medication.

In this report, both patients were treated with mechanical ventilation and, in Case 2, ultimately escalated to ECMO. Literature describing severe and progressive respiratory failure secondary to pulmonary blastomycosis in pediatric patients is limited, including one case report of a child managed with noninvasive ventilation and a case series from Canada that identified three children requiring intubation, all of whom survived treatment [25]. Initiation of ECMO earlier in progressive respiratory failure due to infectious and noninfectious etiologies is associated with an increase in survival [6, 26]. Historically, however, there has been concern for initiating ECMO in patients with fungemia, with concerns for adherence of the organism to the ECMO circuit, thus preventing its eradication [7]. Fungal pneumonia as a contraindication for ECMO has been less clear. Case reports have provided compelling anecdotal evidence that early initiation can prove beneficial in patients with respiratory failure secondary to blastomycosis. A 2016 case series of adults reported four patients with ARDS secondary to blastomycosis treated with ECMO survived with a median of 2 days from ICU admission to initiation of ECMO [16]. Another case series reports survival of four adult patients with ARDS secondary to blastomycosis with ECMO being initiated within 72 h [19]. Literature describing pediatric cases of severe pulmonary blastomycosis treated with ECMO is also limited to reports of cases, but outcomes in these reports have not been favorable. In one case series, a 15-year-old patient with a delayed diagnosis of pulmonary blastomycosis succumbed to the infection after progressing to pARDS requiring ECMO [10]. Another describes a teenager with pulmonary blastomycosis that progressed to death despite ECMO support being initiated 11 days into illness [7]. Another case report describes a 12-year-old with a delayed diagnosis leading to progressive failure requiring ECMO who also succumbed to her infection secondary to irreversible lung damage [8]. Here, we report the first pediatric survivor of severe pulmonary blastomycosis with progressive respiratory failure requiring ECMO. This case adds to the limited but growing body of literature suggesting the potential role of ECMO for respiratory support while awaiting response to antifungals in patients with progressive respiratory failure due to blastomycosis [5–8, 19].
